# Placental phenotype and resource allocation to fetal growth are modified by the timing and degree of hypoxia during mouse pregnancy

**DOI:** 10.1113/JP271057

**Published:** 2015-10-26

**Authors:** J. S. Higgins, O. R. Vaughan, E. Fernandez de Liger, A. L. Fowden, A. N. Sferruzzi‐Perri

**Affiliations:** ^1^ Centre for Trophoblast Research, Department of Physiology, Development and NeuroscienceUniversity of CambridgeCambridgeUK

## Abstract

**Key points:**

- Hypoxia is a major cause of fetal growth restriction, particularly at high altitude, although little is known about its effects on placental phenotype and resource allocation to fetal growth.
- In the present study, maternal hypoxia induced morphological and functional changes in the mouse placenta, which depended on the timing and severity of hypoxia, as well as the degree of maternal hypophagia.
- Hypoxia at 13% inspired oxygen induced beneficial changes in placental morphology, nutrient transport and metabolic signalling pathways associated with little or no change in fetal growth, irrespective of gestational age.
- Hypoxia at 10% inspired oxygen adversely affected placental phenotype and resulted in severe fetal growth restriction, which was due partly to maternal hypophagia.
- There is a threshold between 13% and 10% inspired oxygen, corresponding to altitudes of ∼3700 m and 5800 m, respectively, at which the mouse placenta no longer adapts to support fetal resource allocation. This has implications for high altitude human pregnancies.

**Abstract:**

The placenta adapts its transport capacity to nutritional cues developmentally, although relatively little is known about placental transport phenotype in response to hypoxia, a major cause of fetal growth restriction. The present study determined the effects of both moderate hypoxia (13% inspired O_2_) between days (D)11 and D16 or D14 and D19 of pregnancy and severe hypoxia (10% inspired O_2_) from D14 to D19 on placental morphology, transport capacity and fetal growth on D16 and D19 (term∼D20.5), relative to normoxic mice in 21% O_2_. Placental morphology adapted beneficially to 13% O_2_; fetal capillary volume increased at both ages, exchange area increased at D16 and exchange barrier thickness reduced at D19. Exposure to 13% O_2_ had no effect on placental nutrient transport on D16 but increased placental uptake and clearance of ^3^H‐methyl‐d‐glucose at D19. By contrast, 10% O_2_ impaired fetal vascularity, increased barrier thickness and reduced placental ^14^C‐methylaminoisobutyric acid clearance at D19. Consequently, fetal growth was only marginally affected in 13% O_2_ (unchanged at D16 and −5% at D19) but was severely restricted in 10% O_2_ (−21% at D19). The hypoxia‐induced changes in placental phenotype were accompanied by altered placental insulin‐like growth factor (IGF)‐2 expression and insulin/IGF signalling, as well as by maternal hypophagia depending on the timing and severity of the hypoxia. Overall, the present study shows that the mouse placenta can integrate signals of oxygen and nutrient availability, possibly through the insulin‐IGF pathway, to adapt its phenotype and optimize maternal resource allocation to fetal growth during late pregnancy. It also suggests that there is a threshold between 13% and 10% inspired O_2_ at which these adaptations no longer occur.

## Key points

- Hypoxia is a major cause of fetal growth restriction, particularly at high altitude, although little is known about its effects on placental phenotype and resource allocation to fetal growth.
- In the present study, maternal hypoxia induced morphological and functional changes in the mouse placenta, which depended on the timing and severity of hypoxia, as well as the degree of maternal hypophagia.
- Hypoxia at 13% inspired oxygen induced beneficial changes in placental morphology, nutrient transport and metabolic signalling pathways associated with little or no change in fetal growth, irrespective of gestational age.
- Hypoxia at 10% inspired oxygen adversely affected placental phenotype and resulted in severe fetal growth restriction, which was due partly to maternal hypophagia.
- There is a threshold between 13% and 10% inspired oxygen, corresponding to altitudes of ∼3700 m and 5800 m, respectively, at which the mouse placenta no longer adapts to support fetal resource allocation. This has implications for high altitude human pregnancies.

**Abbreviations**
Akt: protein kinase B
AMPK: adenosine monophosphate‐activated protein kinase
D: day of pregnancy
Db: decidua basalis
FC: fetal capillaries
H: hypoxia
IGF: insulin‐like growth factor
IGF1R: IGF receptor‐β
IR: insulin receptor
Jz: junctional zone
Lz: labyrinthine zone
MBS: maternal blood space
MeAIB: ^14^C‐methyl‐aminoisobutyric acid
MeGlu: ^3^H‐methyl‐d‐glucose
N: normoxic
PF: pair‐fed
PI3K: phosphatidylinositol3‐kinase

## Introduction

Hypoxia is a common complication of pregnancy, occurring in 9–10% of pregnancies at sea level as a result of smoking, anaemia, cord occlusion or poor placental vascularity (Hutter *et al*., 2010), as well as in all pregnancies at high altitude (Zamudio, 2003; Tissot van Patot *et al*., 2010). It is often associated with intrauterine growth restriction, which has immediate adverse consequences for the neonate and also increases adult rates of morbidity and mortality (McMillen & Robinson, 2005; Myatt, 2006; Giussani & Davidge, 2013; Zhang *et al*. 2015). In human populations, birth weight decreases on average by ∼100 g for every 1000 m above sea level, suggesting that fetal growth is highly sensitive to alterations in atmospheric oxygen content (Moore *et al*. 2011). However, the decrement is birth weight is less in multigenerational inhabitants of the elevated climes, such as the Tibetans and Andeans, than in more recent settlers, such as the Han Chinese and Europeans (Giussani *et al*. 2001; Postigo *et al*. 2009; Moore *et al*. 2011; Soria *et al*. 2013). Decrements in fetal growth have also been observed in mice and rats exposed to hypoxic conditions during late pregnancy (Zhou *et al*. 2013; Cuffe *et al*. 2014). By contrast, hypoxia in sheep and guinea pig pregnancies does not necessarily reduce birth weight, even during chronic maternal exposure, although the severity of the hypoxia and the gestational age at onset are both critical (Bacon *et al*. 1984; Jacobs *et al*. 1988; Krebs *et al*. 1996; Penninga & Longo, 1998; Parraguez *et al*. 2005). Taken together, these observations suggest that there are adaptations in materno‐fetal resource allocation during chronic hypoxia that help to maintain fetal growth, although the mechanisms operating *in vivo* remain unknown.

The main determinant of fetal growth is the placental supply of oxygen and nutrients (Burton & Fowden, 2015). Previous studies in rodents and other experimental animals have shown that the placenta responds to environmental cues such as maternal stress, energy intake and dietary composition by adapting its morphological and functional phenotype to optimize fetal growth with respect to the available resource (Fowden *et al*. 2009; Vaughan *et al*. 2012 *a*; Sferruzzi‐Perri *et al*. 2013 *a*). In rodents, there are changes in placental vascularity, barrier thickness, passive diffusion, nutrient transport and nutrient transporter expression in response to maternal dietary perturbations during pregnancy, particularly when placental growth is compromised (Jansson *et al*. 2006; Jones *et al*. 2008; Coan *et al*. 2010, 2011; Rosario *et al*. 2011; Sferruzzi‐Perri *et al*. 2011, 2013 *b*). At high altitude, the human and ovine placenta can adapt morphologically to improve the oxygen diffusion capacity by increasing fetal vascularity and thinning the diffusion barrier between the maternal and fetal circulations (Ali *et al*. 1996; Krebs *et al*. 1996; Mayhew, 1998; Tissot van Patot *et al*. 2003; Parraguez *et al*. 2006). Similar increases in placental vascularity and diffusion capacity have been observed in guinea pigs exposed to hypoxia for most of pregnancy (Bacon *et al*. 1984). In rodents, maternal hypoxia in late pregnancy can either decrease or increase placental vascularity, particularly in the labyrinthine zone (Lz) responsible for nutrient transfer, depending on the severity of the hypoxic insult (Gheorghe *et al*. 2007; Hvizdosova‐Klescova *et al*. 2013; Zhou *et al*. 2013; Cuffe *et al*. 2014). Compared to these morphological changes, however, little is known about any functional adaptations in placental phenotype *in vivo*, notably its nutrient transport capacity, during hypoxic conditions in any species.

One mechanism by which maternal hypoxia may modify placental transport phenotype is by modulating the growth regulatory gene, insulin‐like growth factor (IGF)(i.e. *Igf2*) and the insulin/IGF signalling pathway. In mice, deletion of the placental specific *P0* transcript of the *Igf2* gene prevents the beneficial adaptations in placental morphology and nutrient transport in response to maternal undernutrition (Sferruzzi‐Perri *et al*. 2011; Diaz *et al*. 2014) and deletion of all *Igf2* transcripts impairs passive diffusion of solutes and active amino acid transport across the mouse placenta (Coan *et al*. 2008, 2010, 2011; King *et al*. 2013). In addition, there are placental changes in the insulin/IGF signalling pathway downstream of the receptors that involve phosphatidylinositol 3‐kinase (PI3K) in response to maternal undernutrition or feeding obesogenic diets during pregnancy in mice, sheep and non‐human primates (Zhu *et al*. 2007, 2009; Ma *et al*. 2011; Sferruzzi‐Perri *et al*. 2011, 2013 *b*; Kavitha *et al*. 2014; Lager *et al*. 2014). Hypoxia also regulates the expression of *Igf2* in tissues, including those of the fetus (Feldser *et al*. 1999); however, nothing is known about placental *Igf2* expression or insulin/IGF signalling during hypoxic conditions. Alternatively, hypoxia‐induced adaptation of placental transport phenotype may involve changes in maternal circulating leptin because leptin production is modified by hypoxia, affects the tissue expression of IGF system components and modulates nutrient transport activity in the placenta (Jansson *et al*. 2003; Sferruzzi‐Perri *et al*. 2011; Sferruzzi‐Perri *et al*. 2013 *a*,*b*). Accordingly, in the present study, we tested the hypothesis that maternal hypoxia alters placental transport phenotype in association with changes in maternal leptin and placental insulin/IGF signalling with consequences for fetal growth during late mouse pregnancy. In particular, we investigated the morphological and transport adaptations of the placenta in response to 13% and 10% oxygen in the inspired air for 5 days during the second half of pregnancy relative to normoxic controls in 21% oxygen. This degree of hypoxia is equivalent to altitudes between ∼3700 m and 5800 m at which rodent and human populations live but where habitation becomes progressively sparse (West, 2002; Storz *et al*. 2007).

## Methods

### Animals

**Figure 1:**
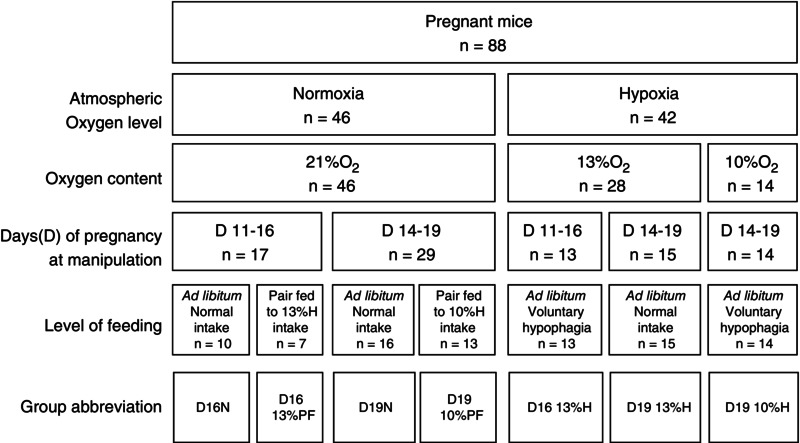
**Schematic diagram of the experimental protocol** Schematic diagram of the experimental protocol and the allocation of pregnant mice between the different treatments, together with the group abbreviations for the treatments used.

All procedures were carried out under the UK Animal (Scientific Procedures) Act 1986. Eighty‐eight virgin C57BL/6 J female mice, aged 6–8 weeks, were housed in groups of two to five per cage under a 12:12 h light:dark cycle at 22°C and were mated overnight with C57BL/6 J males. The presence of a copulatory plug was designated as day (D)1 of pregnancy (term ∼D20.5). All animals had *ad libitum* access to water and food [RM3, energy from fat 11%, protein 26%, carbohydrate 62% (simple sugar 7%), 15.3 MJ kg^−1^, diet code 801066; Special Diet Services, Witham, Essex, UK]. Mated females were weighed daily and the daily consumption of food and water was measured per cage to calculate intake per mouse per day. Pregnant mice were exposed to chronic, normobaric hypoxia for 5 day periods by placing their cages into an isolated PVC chamber (PFI Plastics Ltd, Milton Keynes, UK) in which the oxygen content was reduced to either 13% or 10% by displaced oxygen with nitrogen using a nitrogen generator (N2MID60; Domnick Hunter Ltd, Warwick, UK). The experimental protocol is shown in Fig. 1. Specifically, dams were either exposed to moderate hypoxia (13% O_2_) from D11 to D16 or from D14 to D19 or they were exposed to more severe hypoxia (10% O_2_) from D14 to D19 only (Fig. 1). Exposure to 10% O_2_ from D11 to D16 was not compatible with a viable pregnancy. Control mice remained normoxic at an atmospheric oxygen content of 21% throughout their pregnancies but were housed in the same room as the hypoxic chambers for the corresponding D11 to D16 or D14 to D19 periods of pregnancy (Fig. 1). Relative to the normoxic (N) controls (D16N and D19N), food intake was voluntarily reduced during the 5 day period of hypoxia (H) in dams exposed to 10% O_2_ from D14 to D19 (10% H, 2.2 ± 0.5 g mouse day^−1^, *n* = 8 cages; N, 4.0 ± 0.1 g mouse day^−1^, *n* = 9 cages, *P* < 0.05) and to 13% O_2_ from D11 to D16 (13% H, 3.0 ± 0.3 g mouse day^−1^, *n* = 5 cages; N, 3.9 ± 0.2 g mouse day^−1^, *n* = 5 cages, *P* < 0.05) but not from D14 to D19 (*P* > 0.05). Consequently, pregnant mice were kept in normoxic conditions and pair‐fed (PF) to the intakes of the 13% H and 10% H mice over the corresponding periods of pregnancy as additional normoxic pair‐fed controls (Fig. 1). Pair‐fed mice were housed in the room containing the hypoxia chambers from D11 to D16 or D14 to D19.

### Experimental procedures

Between 08.00 h and 10.00 h on either D16 or D19, dams were anaesthetized with an i.p. injection of fentanyl‐fluanisone and midazolan in sterile water (1:1:2; 10 μg ml^−1^; Janssen Animal Health, High Wycombe, UK). A blood sample was taken from the tail vein to measure haematocrit. As described previously (Sibley *et al*. 2004), unidirectional materno‐fetal clearance of the non‐metabolizable radiolabelled analogues of glucose and an amino acid, ^3^H‐methyl‐d‐glucose (MeGlu, NEN NEC‐377, 2.1 GBq mmol^−1^; Perkin Elmer, Waltham, MA, USA) and ^14^C‐methylaminoisobutyric acid (MeAIB, NEN NEC‐671, 1.86 GBq mmol^−1^; Perkin Elmer), respectively was then measured *in vivo* (D16N, *n* = 7; D16 13% PF, *n* = 7; D16 13% H, *n* = 10; D19N, *n* = 13; D19 13% H, *n* = 14; D19 10% PF, *n* = 12; D19 10% H, *n* = 14). One to four minutes after tracer injection, a cardiac blood sample was collected immediately before killing the anaesthetized dam by cervical dislocation. The average time from tracer administration to cardiac sampling was ∼2 min and did not differ between groups. The blood sample was placed into an EDTA‐coated tube kept at 4°C after measurement of the blood glucose concentration using a hand‐held glucometer (One Touch Ultra; Lifescan, Tunbridge Wells, UK). The uterus was removed from each dam and the numbers of viable and resorbing conceptuses were counted before the fetuses were decapitated. The maternal carcass and individual organs plus each fetus and corresponding placenta were weighed. The placenta with the weight closest to the litter mean was bisected mid‐sagitally and each half was weighed. One half was placed in 4% paraformaldehyde and the other in 4% gluteraldehyde for histological analyses. The second lightest placenta was snap frozen whole and stored at −80°C for subsequent analysis of tracer uptake. The Lz of the second and third heaviest placentae were separated from the endocrine junctional zone (Jz) and snap frozen in liquid nitrogen and stored at −80°C for subsequent analysis of protein and gene expression, respectively. The maternal blood was centrifuged and the plasma stored at −20°C for subsequent biochemical analyses.

### Placental morphometry

Paraformaldehyde‐fixed placental halves were embedded in paraffin wax, exhaustively sectioned at 7 μm and stained with hematoxylin and eosin (D16N, *n* = 7; D16 13% PF, *n* = 5; D16 13% H, *n* = 8; D19N, *n* = 7; D19 13% H, *n* = 7; D19 10% PF, *n* = 7; D19 10% H, *n* = 9). The absolute and percentage volumes of the placental Jz, Lz and the maternal portion of the placenta, the decidua basalis (Db), were determined by point counting with a 10× objective lens using the Computer Assisted Stereological Toolbox (CAST, version 2.0; Olympus, Ballerup, Denmark), as described previously (Coan *et al*. 2004). Gluteraldehyde‐fixed placental halves were embedded in Spurr's epoxy resin and a single, 1 μm mid‐line section was cut and then stained with toluidine blue (D16 all groups *n* = 5, D19N, *n* = 6; D19 13% H, *n* = 4; D19 10% PF, *n* = 6; D19 10% H, *n* = 6). The absolute and percentage volumes of maternal blood spaces (MBS), fetal capillaries (FC) and trophoblast in the Lz, the absolute surface areas of MBS and FC, the total surface area for exchange (averaged surface area of MBS and FC) and the thickness of the interhemal membrane were determined with a 10× objective lens using CAST, as described previously (Coan *et al*. 2004).

### Biochemical analysis

#### Maternal hormone concentration

The concentrations of circulating insulin, IGF‐1 and leptin in maternal plasma were measured in duplicate by an enzyme‐linked immunoabsorbant assay in accordance with the manufacturers’ instructions (Crystal Chem Inc., Downers Grove, IL, USA; R&D Systems, Minneapolis, MN, USA, respectively) from all the groups (D16 all groups, *n* = 5; D19N, *n* = 7; D19 13% H, *n* = 6; D19 10% PF, *n* = 5; D19 10% H, *n* = 7). The intra‐assay coefficients of variation were 11.6%, 3.9% and 4.3% for the insulin, IGF‐1 and leptin assays, respectively.

#### Placental content and transport of MeGlu and MeAIB

Whole placentas and minced fetuses were digested in Biosol (National Diagnostics, Hull, UK) for ≥ 1 week at 55°C. Beta emissions of maternal plasma and of fetal and placental digestates were measured using a 300 SL Liquid Scintillation Counter (LabLogic, Sheffield, UK). Radioactivity in the fetuses, placentas and maternal plasma was used to calculate either placental clearance of MeGlu and MeAIB (μl min^–1^ g^–1^ of placenta) or tracer accumulation expressed (g^–1^ of fetus or placenta), as described previously (Sibley *et al*. 2004).

#### Placental gene expression

Real‐time PCR was used to determine the Lz expression of glucose transporter isoforms (*Slc2a1* and *3*), System A amino acid transporters (*Slc38a1, 2* and *4*), all *Igf2* transcripts and the Lz‐specific *Igf2* transcript, *Igf2P0*. RNA was extracted from each placental Lz using the RNeasy Plus Mini Kit (Qiagen, Crawley, UK: D16N, *n* = 6; D16 13% PF, *n* = 5; D16 13% H, *n* = 7; D19N, *n* = 7; D19 13% H, *n* = 7; D19 10% PF, *n* = 7; D19 10% H, *n* = 6). Extracted RNA (2.5 μg) was reverse transcribed to cDNA on a DNA Engine Thermal Cycler (MyCycler; Bio‐Rad, Hercules, CA, USA) using Multiscribe Reverse Transcriptase (Applied Biosystems, Warrington, UK). Samples were analysed in duplicate on a 7500 Fast Real‐Time PCR instrument (Applied Biosystems) using TaqMan assays for *Slc2a1* (Mm00441473_m1), *Slc2a3* (Mm00441483_m1), *Slc38a1* (Mm00506391_m1), *Slc38a2* (Mm00628416_m1), *Slc38a4* (Mm00459056_m1), *Igf2* (Mm00439564_m1)and *Igf2p0* (forward primer CCGAGGCCTGTACCACCTA, reverse primer CCTCGGCTCAGACCTCAGTA, FAM CCGAGGCCTCTGCCACC), as reported previously (Coan *et al*. 2010). Using the ΔΔCt method (Schmittgen & Livak, 2008), the expression of the genes of interest in each sample was normalized to the expression of *Tbp* (Mm01277045_m1), which did not vary between experimental groups, and then expressed relative to the normoxic, *ad libitum* fed, controls (N group) at each age. The Taqman primers/probes for all genes, including the housekeeper were of similar efficiency at ∼100%.

#### Placental protein expression

Proteins were extracted from the Lz (all groups, *n* = 5) in lysis buffer containing 20 mm Tris (pH 7.5), 150 mm NaCl, 1 mm Na_2_EDTA, 1 mm EGTA, 1% Triton X‐100, 2.5 mm sodium pyrophosphate, 1 mm β‐glycerolphosphate, 1 mm Na_3_VO_4_ and complete miniproteases inhibitor cocktail (Roche Diagnostics, Burgess Hill, UK). Lysate protein concentrations were determined using a Bicinchoninic acid assay (Sigma‐Aldrich, St Louis, MO, USA). Equivalent lysate protein concentrations (50 μg) were resolved by SDS‐PAGE and transferred onto nitrocellulose membranes. Ponceau‐S staining of membranes confirmed equal protein loading between samples. Membranes were probed with antibodies against insulin receptor‐β (IR; Santa Cruz Biotechnology, Dallas, TX, USA), type 1 IGF receptor‐β (IGF1R; Santa Cruz Biotechnology), p85α (Millipore, Billerica, MA, USA), p110α, p110β, Akt‐T308‐P, Akt‐S473‐P and total Akt (Cell Signaling Technology, Beverly, MA, USA) and bands depicting antibody‐bound proteins were visualized on photographic film. Band intensities were assessed by densitometry using ImageJ (NIH, Bethesda, MD, USA).

### Statistical analysis

All data are presented as the mean ± SEM and were analysed using SPSS, version 21.0 (IBM Corp., Armonk, NY, USA). *P* < 0.05 was considered statistically significant. Separate statistical comparisons were made at the two stages of pregnancy and with the degree of hypoxia exposure. At D16 of pregnancy, the three experimental groups were compared by one‐way ANOVA with Bonferroni *post hoc* tests (D16N, D16 13% H and D16 13% PF). On D19, normoxic (N) and 13% H groups were compared using Student's unpaired *t* test, whereas normoxic (N), 10% PF and 10% H groups were compared by one‐way ANOVA with Bonferroni *post hoc* tests. For fetal and placental biometry, placental clearance and accumulation of MeGlu and MeAIB and fetal accumulation of these tracers, statistical tests were performed using litter means.

## Results

### Biometry

#### 13% O_2_

Maternal exposure to 13% O_2_ for 5 days had little effect on total body weight, hysterectomized weight, non‐uterine weight gain or on the weight of the individual maternal organs compared to normoxic *ad libitum* fed controls at either stage of pregnancy (see Supporting information, Table S1). However, hysterectomized weight was greater in mice exposed to 13% O_2_ from D11 to D16 than in D16 normoxic dams pair‐fed to the intake of 13% H dams (see Supporting information, Table S1). Moreover, non‐uterine weight gain was reduced in 13% PF dams, relative to the D16N controls (see Supporting information, Table S1). Exposure to 13% O_2_ did not affect fetal or placental weights at D16, relative to both *ab libitum* and pair‐fed controls (Table 1). On D19, fetal weight was ∼5% less in 13% H than normoxic dams, with no significant difference in placental weight (Table 1).

**Table 1:** Conceptus biometry and morphology of the placenta on D16 and D19 of pregnancy in normoxic dams (21% atmospheric O_2_ content); hypoxic dams exposed for 5 days either to 13% O_2_ from D11 to D16 or D14 to D19 or to 10% O_2_ from D14 to D19; and normoxic dams pair‐fed to the food intake of the 13% H dams from D11 to D16 or of the 10% H dams from D14 to D19

	**D16**			**D19**			
	**Normoxia**	**13% PF**	**13% H**	**13% H**	**Normoxia**	**10% PF**	**10% H**
**Conceptus weight (mg)**							
Fetus	402 ± 19	403 ± 9	409 ± 17	**1111** ± 16*	**1173** ± **11^a^**	**1068** ± **21^b^**	**930** ± **23^c^**
Placenta	100.3 ± 3.3	100.3 ± 4.3	103.5 ± 2.1	90.0 ± 1.8	87.5 ± 2.6	84.5 ± 2.6	91.3 ± 1.8
F:P	4.0 ± 0.1	4.1 ± 0.2	3.9 ± 0.2	**12.5** ± 0.3*	**13.6** ± **0.3^a^**	**12.9** ± **0.5^a^**	**10.3** ± **0.3^b^**
Volume (mm^3^)							
Lz	**39.9** ± **1.9^a^**	**44.0** ± **2.0^ab^**	**47.0** ± **1.8^b^**	43.7 ± 1.2	43.5 ± 1.7	46.4 ± 0.1	44.3 ± 0.7
Jz	47.0 ± 3.0	39.7 ± 4.4	44.9 ± 2.7	33.4 ± 1.7	**31.5** ± **1.9^ab^**	**25.9 ± 1.9^a^**	**37.1** ± **1.8^b^**
Db	13.9 ± 0.7	17.3 ± 2.7	13.2 ± 2.2	9.7 ± 1.2	11.2 ± 1.7	10.5 ± 0.1	9.2 ± 0.7
MBS	**8.1** ± **0.6^a^**	**11.8** ± **0.7^b^**	**10.5** ± **0.4^b^**	10.3 ± 1.1	**10.5** ± **1.0^ab^**	**12.5** ± **0.8^a^**	**9.6** ± **0.6^b^**
FC	5.5 ± 1.0	5.1 ± 0.4	5.5 ± 0.3	**14.2** ± 1.0*	**10.1** ± **0.7**	10.4 ± 0.9	8.3 ± 1.1
Trophoblast	**26.2** ± **1.5**	**28.5** ± **1.8**	**32.6** ± **1.7**	21.7 ± 1.1	23.1 ± 0.7	24.6 ± 1.9	26.8 ± 2.4
**Volume (%)**							
Lz	39.7 ± 1.6	44.0 ± 3.0	44.8 ± 1.7	50.3 ± 1.3	**50.5** ± **1.8^ab^**	**56.0 1.3^a^**	**48.9** ± **0.8^b^**
Jz	46.5 ± 1.6	39.1 ± 2.3	42.7 ± 2.5	38.5 ± 1.4	**36.5** ± **1.5^ab^**	**31.3** ± **2.1^a^**	**40.9** ± **1.6^b^**
Db	13.9 ± 0.9	16.9 ± 1.8	12.5 ± 2.0	11.2 ± 1.3	13.0 ± 1.8	12.7 ± 1.3	10.3 ± 0.8
MBS	20.5 ± 1.5	23.7 ± 1.9	20.6 ± 1.2	22.3 ± 1.4	23.8 ± 1.1	26.5 ± 2.0	22.5 ± 1.7
FC	13. 6 ± 1.7	12.2 ± 1.3	11.1 ± 0.6	**30.6** ± 1.8*	**23.1** ± **0.6^a^**	**21.8** ± **1.0^ab^**	**17.5** ± **1.8^b^**
Trophoblast	66.0 ± 1.0	64.1 ± 2.0	68. 3 ± 1.5	**47.2** ± 1.6*	**53.1** ± **1.6^ab^**	**51.7** ± **1.9^a^**	**60.0** ± **2.5^b^**
**Surface area (cm^2^)**							
MBS	25.7 ± 1.7	31.8 ± 1.5	34.5 ± 4.2	29.7 ± 1.0	**27.1** ± **1.0^a^**	**33.4** ± **1.9^b^**	**23.6** ± **2.0^a^**
FC	**24.0** ± **1.3**	**35.2** ± **4.0**	**32.8** ± **3.2**	26.5 ± 2.0	24.9 ± 2.3	26.2 ± 2.1	19.8 ± 2.0
Total SA for exchange	**24.9** ± **1.5^a^**	**33.5** ± **1.9^b^**	**33.7** ± **1.4^b^**	28.1 ± 2.1	**26.0** ± **1.6^ab^**	**29.8** ± **1.7^a^**	**21.7** ± **1.8^b^**
Th (μm)	3.27 ± 0.14	2.92 ± 0.13	3.12 ± 0.19	**2.57** ± 0.07*	**2.85** ± **0.07^a^**	**2.53** ± **0.03^b^**	**3.08** ± **0.06^c^**

Data are the mean ± SEM. For biometry: D16N, *n* = 10; D16 13% H, *n* = 13; D16 13% PF, *n* = 7: D19N, *n* = 16; D19 13% H, *n* = 15; D19 10% H, *n* = 14; D19 10% PF, *n* = 13. For placental morphology: D16N, *n* = 5–7; D16 13% H, *n* = 5–8; D16 13% PF, *n* = 5: D19N, *n* = 6–7; D19 13% H, *n* = 4–7; D19 10% H, *n* = 6–9; D19 10% PF, *n* = 6–7. Significant differences between groups are indicated in bold. On D16, values with different superscript letters are significantly different from each other by one‐way ANOVA (*P* < 0.05) and the Bonferroni *post hoc* test (*P* < 0.05). On D19, an asterisk (*) indicates that 13% H is significantly different from D19N by Student's *t* test (*P* < 0.04), whereas values for the D19N, D19 10% H and D19 10% PF groups with different superscript letters are significantly different from each other by one‐way ANOVA (*P* < 0.05) and the Bonferroni *post hoc* test (*P* < 0.05). F:P, fetal weight to placental weight ratio; SA, surface area; Th, harmonic mean thickness; Total SA for exchange, average of MBS and FC surface areas.

#### 10% O_2_

Maternal exposure to the more severe hypoxia of 10% inspired O_2_ from D14 to D19 reduced total body weight, hysterectomized weight and non‐uterine weight gain at D19 compared to D19N controls only, with intermediate values in D19 10% PF group (see Supporting information, Table S1). Weights of the maternal liver and retroperitoneal fat were reduced in 10% hypoxic mice relative to the D19N controls, although these values were similar to the pair‐fed group (see Supporting information, Table S1). Exposure to 10% O_2_ also reduced fetal weight by 21%, compared to the D19N group (Table 1). Pair‐feeding also reduced fetal weight by 10%, compared to the D19N group (Table 1). Placental weight was similar in the 10% H, D19N and 10% PF groups (Table 1).

### Maternal metabolite and hormone concentrations

#### 13% O_2_

Maternal exposure to 13% O_2_ reduced maternal blood glucose concentration on D16 but not D19 of pregnancy, relative to their corresponding normoxic *ad libitum* and pair‐fed control groups (see Supporting information, Table S1). Maternal plasma concentrations of insulin, IGF‐1 and leptin were not affected by maternal exposure to 13% O_2_ at either stage of pregnancy (see Supporting information, Table S1). However, D16 13% PF mice had significantly lower plasma leptin concentrations than the D16N or D16 13% H dams (see Supporting information, Table S1). Maternal haematocrit was significantly higher in hypoxic than normoxic dams at both stages of pregnancy (see Supporting information, Table S1).

#### 10% O_2_

Maternal exposure to 10% O_2_ also significantly increased maternal haematocrit on D19, relative to normoxic dams (see Supporting information, Table S1). On D19, maternal concentrations of blood glucose and of plasma IGF‐1 and insulin were similar in the three groups (see Supporting information, Table S1). Consistent with maternal body fat content, the plasma leptin concentration in D19 10% H dams was half that measured in D19N dams and similar to the D19 10% PF dams (see Supporting information, Table S1).

### Placental morphology

#### 13% O_2_

On D16, the absolute volume of the maternal blood spaces and the total Lz surface area for exchange in 13% H dams were significantly greater than D16N but similar to D16 13% PF dams (Table 1). The absolute volume of the Lz was also greater in 13% H than D16N dams, with intermediate values in the 13% PF mice (Table 1). There was also an overall effect of treatment on the absolute volume of the trophoblast and the surface area of the fetal capillaries in the Lz on D16 (Table 1). On D19, both the absolute and percentage volume of the fetal capillaries were greater in 13% H than normoxic dams (Table 1). In addition, maternal exposure to 13% O_2_ from D14 to D19 significantly reduced the harmonic mean thickness of the interhemal membrane (Table 1), which is inversely related to the ease with which oxygen crosses the placental barrier. None of the other morphological parameters measured were affected by maternal exposure to 13% O_2_ or pair‐feeding on D16 or D19 (Table 1).

#### 10% O_2_

On D19, the majority of the significant differences in placental morphology were seen between the hypoxic and pair‐fed dams, with intermediate values in the D19N group (Table 1). Although not different compared to the D19N group, the absolute and percentage volume of the Jz were greater, whereas the percentage Lz volume, absolute volume and surface area of the maternal blood spaces and the total surface area for exchange were less in the D19 10% H than the D19 10% PF group (Table 1). However, the percentage volume of the Lz occupied by fetal capillaries was significantly less in D19 10% H than D19N dams, whereas the harmonic mean thickness of the interhemal membrane was significantly more in D19 10% H dams than in both D19N and D19 10% PF groups (Table 1).

### Placental glucose and amino acid transport

#### 13% O_2_

**Figure 2:**
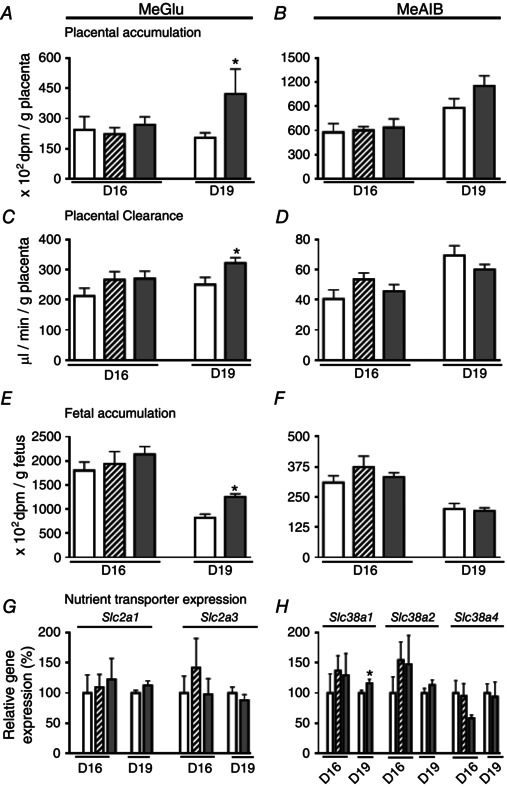
**Placental transport following exposure to 13% maternal inhalation hypoxia** Materno‐fetal transport of MeGlu and MeAIB and Lz gene expression of glucose (*Slc2a1* and *Slc2a3*) and System A amino acid transporters (*Slc38a1*, *Slc38a2* and *Slc38a4*) on D16 and D19 of pregnancy following exposure to 13% O_2_ inspired air for 5 days from D11 to D16 or D14 to D19 or pair‐feeding normoxic animals to the food intake of mice in 13% O_2_ from D11 to D14. Data are the mean ± SEM of placental accumulation of MeGlu (*A*) and MeAIB (*B*), placental clearance of MeGlu (*C*) and MeAIB (*D*), fetal accumulation of MeGlu (*E*) and MeAIB (*F*) and Lz gene expression of glucose transporters (*G*) and System A amino acid amino acid transporters (*H*). Normoxic *ad libitum* fed, white columns, D16N, *n* = 6–7, D19N, *n* = 7–13; pair‐fed normoxic animals, striped columns, D16 13% PF, *n* = 5–7; 13% O_2_ hypoxic, D16 13% H, *n* = 7–10 and D19 13% H, *n* = 7–13, grey columns. An asterisk denotes significant difference from the normoxic *ad libitum* fed (N) group at the same age. *P < 0.05 (Student's *t* test).

Maternal exposure to 13% O_2_ from D11 to 16 did not affect placental accumulation, unidirectional materno‐fetal clearance or fetal accumulation of either MeGlu or MeAIB on D16, relative to the D16N and D16 13% PF dams (Fig. 2 *A–F*). There were also no differences in gene expression of the glucose (*Slc2a1* and *Slc2a3*) and System A amino acid transporters (*Slc38a1*, *Slc38a2* and *Slc38a4*) in the placental Lz between the three groups of dams at D16 (Fig. 2 *G* and *H*). By contrast, maternal exposure to 13% O_2_ later in pregnancy significantly increased placental uptake, unidirectional materno‐fetal clearance and fetal accumulation of MeGlu, relative to the D19N controls (Fig. 2 *A*, *C* and *E*). This occurred in the absence of any significant change in *Slc2a1* or *Slc2a3* expression in the placental Lz (Fig. 2 *G*). Placental accumulation, unidirectional materno‐fetal clearance and fetal accumulation of MeAIB on D19 were unaffected by maternal exposure to 13% O_2_ from D14 to D19 (Fig. 2 *B*, *D* and *F*), although *Slc38a1* expression was greater in the placental Lz of 13% H than D19N dams (Fig. 2 *H*).

#### 10% O_2_

**Figure 3:**
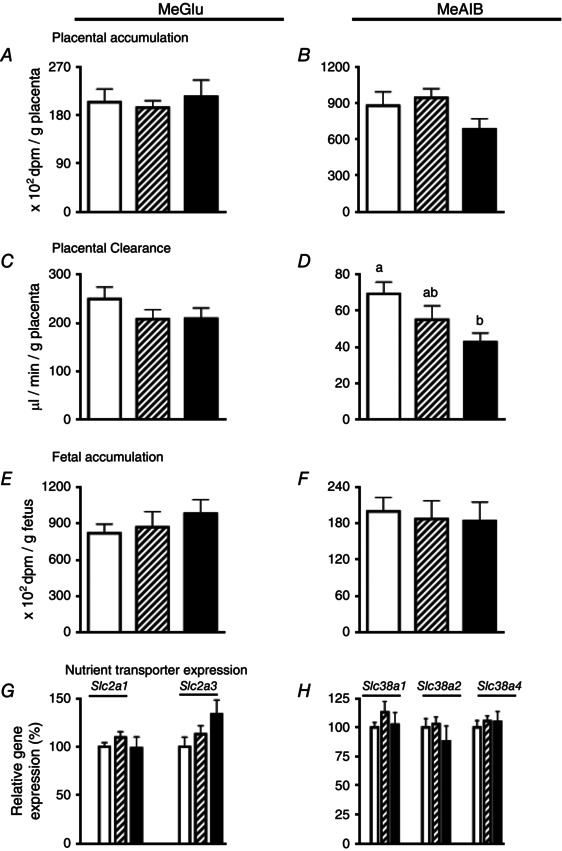
**Placental transport following exposure to 10% maternal inhalation hypoxia** Materno‐fetal transport of MeGlu and and MeAIB and Lz gene expression of glucose (*Slc2a1* and *Slc2a3*) and System A amino acid transporters (*Slc38a1*, *Slc38a2* and *Slc38a4*) on D19 of pregnancy following exposure to 10% O_2_ inspired air for 5 days from D14 to D19 or pair‐feeding normoxic dams to the food intake of the hypoxic animals from D14 to D19. Data are the mean ± SEM of placental accumulation of MeGlu (*A*) and MeAIB (*B*), placental clearance of MeGlu (*C*) and MeAIB (*D*), fetal accumulation of MeGlu (*E*) and MeAIB (*F*) and Lz gene expression of glucose transporters (*G*) and System A amino acid transporters (*H*). Normoxic *ad libitum* fed, white columns, D19N, *n* = 7–13; pair‐fed normoxic animals, striped columns, D19 10% PF, *n* = 7–12; 10% O_2_ hypoxic, black columns, D19 10% H, *n* = 6–14. Columns with different superscript letters are significantly different from each other *P* < 0.05 (one‐way ANOVA with Bonferroni *post hoc* tests). The D19N values are identical to those shown in Fig. 2.

Maternal exposure to 10% O_2_ from D14 to D19 did not affect placental accumulation, unidirectional materno‐fetal clearance or fetal accumulation of MeGlu on D19, relative to both the D19N and D19 10% PF groups (Fig. 3 *A*, *C* and *E*). There were also no significant differences in *Slc2a1* or *Slc2a3* expression in the placental Lz between the three groups of dams at D19 (Fig. 3 *G*). By contrast, placental MeAIB clearance was 30–40% less in hypoxic dams than in the normoxic *ad libitum* fed group at D19, with intermediate values in the pair‐fed group (Fig 3 *D*). Maternal exposure to 10% O_2_ from D14 to D19 did not alter placental Lz expression of the *Slc38a* genes (Fig. 3 *H*).

### *Igf2* gene expression and insulin‐IGF signalling in the Lz of the placenta

#### 13% O_2_

**Figure 4:**
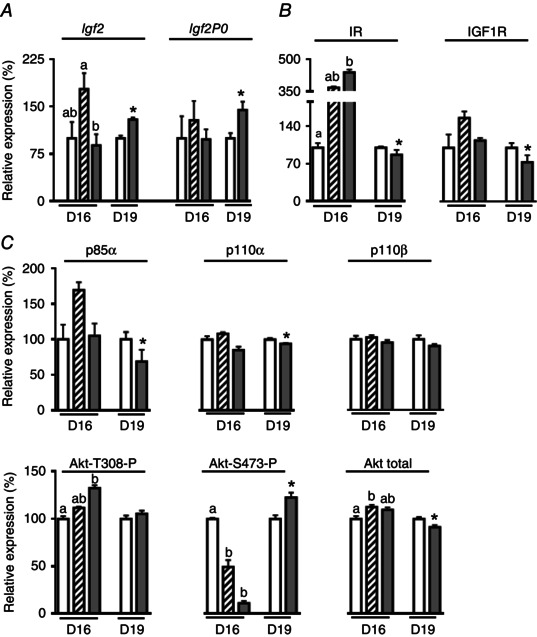
**Placental insulin/IGF signalling following exposure to 13% maternal inhalation hypoxia** Labyrinthine expression of *Igf2* (*A*) and components of the insulin‐IGF signalling pathway (*B* and *C*) on D16 and D19 of pregnancy following exposure to 13% inspired O_2_ for 5 days from D11 to D16 or D14 to D19 or pair‐feeding normoxic mice to the food intake of the hypoxic animals from D11 to D16. Data are the mean ± SEM. Normoxic *ad libitum* fed, white columns, D16N, *n* = 5–6, D19N, *n* = 5–9; pair‐fed animals, striped columns, D16 13% PF, *n* = 5–6; 13% O_2_ hypoxic, D16 13% H, *n* = 5–6 and D19 13% H, *n* = 5–6, grey columns. On D16, columns with different superscript letters are significantly different from each other *P* < 0.05 (one‐way ANOVA with Bonferroni *post hoc* tests). On D19, an asterisk denotes a significant difference from the normoxic *ad libitum* fed (N) group at the same age. *P < 0.05 (Student's *t* test).

Maternal exposure to 13% O_2_ from D11 to D16 did not affect Lz expression of either total *Igf2* or the placental‐specific transcript, *Igf2P0*, on D16, relative to D16N controls (Fig. 4 *A*). However, total *Igf2* expression in the Lz was less in the 13% H than 13% PF dams at D16 (Fig. 4 *A*). Maternal exposure to 13% O_2_ from D11 to D16 significantly increased Lz abundance of IR and Akt‐T308P relative to D16N dams, with intermediate values in the D16 13% PF group (Fig. 4 *B* and *C*). By contrast, Lz abundance of Akt‐S473‐P at D16 was reduced by hypoxia compared to *ad libitum* but not pair‐fed, normoxic animals (Fig. 4 *C*). Although the total Lz abundance of Akt in D16 13% H dams did not differ from either 13% N or 13% PF values, it was more abundant in the 13% PF group than in the D16N group (Fig. 4 *C*). The Lz abundances of IGF1R, p85α, p110α and p110β were similar in all groups at D16 (Fig. 4). By contrast to the findings on D16, maternal exposure to13% O_2_ from D14 to D19 increased Lz expression of both total *Igf2* and *Igf2P0* relative to the D19N (Fig. 4 *A*). These changes were accompanied by a significant increase in Akt‐S473‐P abundance and significant reductions in the abundances of IR, IGF1R, p85α, p110α and Akt total in the Lz of D19 13% H relative to normoxic dams (Fig. 4 *B* and *C*). The abundance of p110β and Akt‐T308‐P was unaffected by maternal hypoxia at D19 (Fig. 4).

#### 10% O_2_

**Figure 5:**
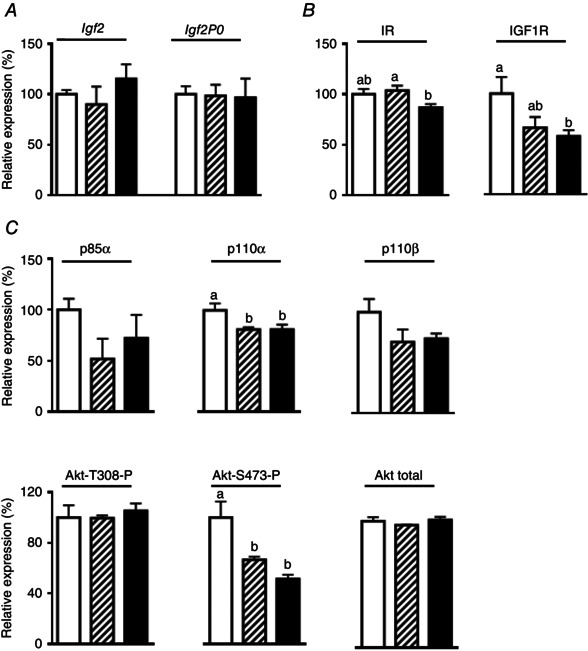
**Placental insulin/IGF signalling following exposure to 10% maternal inhalation hypoxia** Labyrinthine expression of *Igf2* (*A*) and components of the insulin‐IGF signalling pathway (*B* and *C*) on D19 of pregnancy following exposure to 10% inspired O_2_ for 5 days from D14 to D19 or pair‐feeding normoxic mice to the food intake of the hypoxic animals from D14 to D19. Data are the mean ± SEM. Normoxic *ad libitum* fed, white columns, D19N, *n* = 5–9; pair‐fed animals, striped columns, D19 10% PF, *n* = 5–8; 10% hypoxic, black columns, D19 10% H, *n* = 5–9. Columns with different superscript letters are significantly different from each other *P* < 0.05 (one‐way ANOVA with Bonferroni *post hoc* tests).

On D19, the expression of total *Igf2* and *Igf2P0* in the placental Lz was unaffected by maternal exposure to 10% O_2_ or pair‐feeding (Fig. 5 *A*). Maternal exposure to 10% O_2_ from D14 to D19 significantly decreased Lz abundance of IGF1R, p110α and Akt‐S473‐P relative to D19N but not D19 10% PF dams (Fig. 5 *B* and *C*). In addition, IR abundance was less in D19 10% H than D19 10% PF dams, although neither of these values differed significantly from that seen in the D19N group (Fig. 5B). The Lz abundance of p85α, p110β, Akt‐T308‐P and Akt total was similar in the three D19 groups (Fig. 5).

## Discussion

The present study demonstrates that maternal inhalation hypoxia modifies placental phenotype and maternal resource allocation to fetal growth. The placental changes were both morphological and functional in origin and occurred without any change in placental weight. They also were accompanied by altered expression of the *Igf2* gene and proteins in the insulin‐IGF signalling pathway in the Lz of the placenta. The specific nature of these phenotypical changes and their effect on fetal growth depended on the timing and severity of the hypoxic insult and also, in some instances, reflected the concomitant maternal hypophagia. With exposure to 13% O_2_, the placental adaptations in morphology and transfer of glucose and amino acid were largely beneficial to fetal nutrient acquisition and were associated with only a small (5%) restriction in fetal growth at D19. By contrast, maternal exposure to 10% O_2_ during late gestation had detrimental consequences for placental amino acid transfer and materno‐fetal resource allocation, with the result that fetal growth at D19 was reduced by 21%. Taken together, these findings indicate that there is a threshold between 13% and 10% maternal inspired oxygen at which the hypoxic mouse dam and placenta can no longer adapt to support growth of the fetus.

The lack of effect of hypoxia on placental weight in the present study is consistent with previous findings in rats and mice exposed to short periods of hypoxia in late pregnancy (Lueder *et al*. 1995; Saker *et al*. 1999; Cuffe *et al*. 2014). Amongst species, more consistent reductions in placental weight are seen in response to undernutrition, particularly when this occurs throughout most of pregnancy (Rosso, 1977; Dwyer & Stickland,

1992), (Malandro *et al*. 1996; Woodall *et al*. 1996; Fernandez‐Twinn *et al*. 2003; Jansson *et al*. 2006; Mogami *et al*. 2009; Coan *et al*. 2010, 2011; Sferruzzi‐Perri *et al*. 2011). However, in the present study, the relatively short period of undernutrition induced by pair‐feeding in late pregnancy did not affect placental weight. Overall, the observations of the present study suggest that gross growth of the mouse placenta exhibits a degree of resilience to short‐term hypoxic and nutritional insults once it has formed. However, there were ultrastructural changes in the placenta in response to both hypoxia and the undernutrition of pair‐feeding with impacts on placental transport of specific nutrients and, hence, fetal growth.

In dams exposed to 13% O_2_, the Lz volume was increased on D16 in association with expansion of the maternal blood spaces, the fetal capillary surface area and the total surface area for exchange, although there was no change in the thickness of the placental interhaemel membrane at this age. Similar ultrastructural changes were seen in the dams pair‐fed to the reduced intake of the 13% H dams, which suggests that the adaptations in placental morphology at D16 were driven largely by maternal undernutrition. By contrast, exposure to 13% O_2_ in late pregnancy increased fetal vascularity rather than maternal blood space volumes and reduced the thickness of the interhemal membrane at D19, consistent with morphological changes seen in the term placenta of other species exposed to equivalent degrees of hypoxia (Bacon *et al*. 1984; Jacobs *et al*. 1988; Krebs *et al*. 1996; Mayhew, 1998; Parraguez *et al*. 2006). Although the specific morphological adaptations of the 13% H mouse placenta differed at the two stages of pregnancy, their net outcome was similar with respect to increasing the placental capacity for transport of nutrients and O_2_ to the fetus (Fowden *et al*. 2006, 2009). This may explain, in part, the lack of effect of moderate hypoxia at 13% O_2_ on feto‐placental growth at D16 and its relatively minor effect on fetal body weight at D19. In comparison, exposure to 10% O_2_ had detrimental effects on placental morphology, with decreased fetal vascularity and increased barrier thickness at D19, consistent with the more severe degree of fetal growth restriction in this group. These morphological changes appear to have been a result of hypoxia alone because they were not seen in dams pair‐fed to the reduced food intake of the D19 10% H group. Indeed, in contrast to more prolonged maternal undernutrition (Coan *et al*. 2010), the short period of nutrient restriction induced by pair‐feeding caused changes in placental morphology that are probably more beneficial than detrimental to fetal nutrient delivery. As a result, the degree of fetal growth restriction in the hypophagic dams exposed to 10% O_2_ was more than twice that seen in the normoxic pair‐fed dams at D19.

In addition to the changes in placental morphology induced by maternal hypoxia, there were also functional and metabolic adaptations in the placenta with consequences for resource allocation, particularly at D19. Exposure to 13% O_2_ from D14 to D19 increased placental uptake and clearance of MeGlu and its accumulation in the fetus, which suggests that glucose becomes a more important metabolic substrate in feto‐placental tissues when oxygen availability is limited near term. Indeed, increased glycolytic use of glucose by the placenta would spare O_2_ for onward passage to the fetus, in line with suggestions made previously for the high altitude human placenta (Illsley *et al*. 2010). As well as reducing the placental O_2_ requirement, increasing placental glycolysis during hypoxia would help to maintain an ATP supply for the active transport of amino acids, which is consistent with the finding in the present study that placental MeAIB clearance at D19 was unaffected by maternal exposure to 13% O_2_. In addition, the increased transplacental supply of glucose in the D19 13% H group may have promoted glycolytic metabolism in the fetuses and hence maintained a supply of ATP for fetal growth and metabolism in the face of a limited capacity for oxidative metabolism. Certainly, previous studies in pregnant rats have shown increased glucose uptake and lactate production by the fetuses during hypoxic conditions, in association with normal growth of key fetal tissues, such as the heart and brain (Lueder *et al*. 1995). Because fetal growth in rodents is dependent on placental System A amino acid transport and is more closely correlated to placental amino acid than glucose delivery in late gestation (Cramer *et al*. 2002; Coan *et al*. 2011), the adaptations in the transport and metabolic characteristics of the D19 13% H placenta will also help to minimize the degree of fetal growth restriction near term.

By contrast, the more severe insult of 10% O_2_ had no effect on placental glucose uptake or clearance but reduced placental MeAIB clearance in line with the compromised placental morphology and more severe fetal growth restriction. Similar reductions in placental System A activity have been reported in human placental cells cultured *in vitro* under severe hypoxic conditions (Nelson *et al*. 2003; Kleppa *et al*. 2014) and in human placental samples from compromised pregnancies with fetal growth restriction (Glazier *et al*. 1997; Jansson *et al*. 1998, 2002; Cetin, 2003). Comparison with the D19 10% PF group suggests that hypoxia and undernutrition contributed equally to the reduced placental MeAIB clearance in the hypophagic D19 10% H group. In previous studies on mice, placental MeAIB transport was increased near term in response to longer term maternal undernutrition and reduced by mild protein restriction throughout pregnancy (Coan *et al*. 2010, 2011; Sferruzzi‐Perri *et al*. 2011; Ganguly *et al*. 2012). This suggests that adaptation of the placental System A amino acid transport system depends on the specific maternal environment and the timing of the environmental challenge. In the present study, both exposure to 10% O_2_ and pair‐feeding altered maternal body composition and reduced maternal adiposity and circulating leptin concentrations at D19. This hypoleptinaemia may have accounted for the nutritional contribution to reduced placental MeAIB clearance because leptin is known to stimulate amino acid uptake in the human placenta (Jansson *et al*. 2003). Other maternal hormones, such as corticosterone, may be involved in the hypoxic contribution to placental MeAIB transport because corticosterone reduced System A activity and was elevated in concentration by short term maternal exposure to 12% O_2,_ without hypophagia, in mice during late pregnancy (Vaughan *et al*. 2012 *b*; Cuffe *et al*. 2014). Alternatively, O_2_ levels may be sufficiently limited at 10% O_2_ to compromise ATP availability for the active transport of amino acids across the placenta via System A transporters. Consistent with this, recent work has shown increased activation of the energy sensor, adenosine monophosphate‐activated protein kinase (AMPK), in the placenta and uterine vessels of mouse dams exposed to 10% O_2_ hypoxia for the same period in pregnancy (Skeffington *et al*. 2016). Enhanced AMPK activation may prevent further energy depletion and optimize utero‐placental blood flow in the prevailing hypoxic environment. However, the restriction in fetal growth as a result of both reduced maternal O_2_ supply and placental amino acid delivery may have reduced the fetal signals of nutrient demand that are assumed to contribute to environmental regulation of placental transport when O_2_ is not so severely limited (Burton & Fowden, 2012). Whatever the cause, the reduced amino acid supply to fetuses of D19 10% H dams will have spared amino acids for maternal use with potential benefits to maternal fitness, given the hypophagia and reduced accumulation of maternal mass during late pregnancy.

The changes in placental phenotype induced by maternal hypoxia may be partly a result of alterations in *Igf2* gene expression and changes in insulin‐IGF signalling in the Lz, even though maternal concentrations of insulin and IGF‐I were unaffected by hypoxia. Previous studies have shown that the *Igf2* gene is essential for placental adaptation to undernutrition in late gestation and influences placental vascularity, barrier thickness and nutrient transport during normal development (Constancia *et al*. 2005; Coan *et al*. 2008, 2010; Sferruzzi‐Perri *et al*. 2011). The disparity of the placental response to moderate and severe hypoxia at D19 may therefore relate to the finding that Lz abundance of the total and placental specific *Igf2* transcripts was increased in the D19 13% H dams but not in the D19 10% H group. At D16, the changes in placental Lz morphology in 13% O_2_ were not associated specifically with altered *Igf2* gene expression but were accompanied by increased IR abundance, which may reflect an attempt to increase Lz sensitivity to the anabolic actions of insulin and IGF (Sferruzzi‐Perri *et al*. 2010, 2013 *a*). This is consistent with the greater Lz volume and total exchange area in these D16 13% H placentas, together with an increased Lz abundance of Akt‐T308‐P, the residue on Akt that is phosphorylated in response to insulin via PI3K (Vadlakonda *et al*. 2013). By contrast, there were decreases in Lz abundance of IR, IGF1R and specific PI3K subunits on D19, in response to 13% O_2_ in late gestation. Because these changes were also seen in the D19 10% H dams, they suggest that anabolic activity mediated by PI3K and the upstream elements of the insulin‐IGF signalling pathway were down‐regulated by oxygen deprivation, irrespective of its severity. However, the effect on the Lz insulin‐IGF pathway downstream of PI3K differed with the degree of hypoxia, with increased Akt‐S473‐P in the D19 13% H group but decreased phosphorylation of this site in both the D19 10% H and D19 10% PF dams. Because Lz abundance of Akt‐S473‐P was also decreased in the D16 13% H and D16 13% PF groups, phosphorylation of Akt‐S473 in the Lz appears to be decreased by undernutrition and increased by hypoxia alone. Consequently, there were changes in the ratio of Akt‐S473‐P to Akt‐T308‐P in all the hypoxic and pair‐fed dams relative to their respective normoxic *ad libitum* fed controls, irrespective of the stage of pregnancy. Because the two Akt phosphorylation sites are regulated independently and may control different cellular processes downstream of PI3K (Vadlakonda *et al*. 2013), the observations made in the present study suggest that Akt phosphorylation may be a key process in the environmental regulation of placental phenotype, particularly because genetic deletion of Akt is also known to affect the vascularity and thickness of the exchange barrier in the mouse placenta (Yang *et al*. 2003). Indeed, changes in the Akt‐S473‐P to Akt‐T308‐P ratio have been observed previously in the mouse placenta in response to maternal dietary and endocrine manipulations, as well as in human placenta at high altitude and in human placental cell lines cultured under hypoxic conditions (Yung *et al*. 2008; Zhu *et al*. 2009; Sferruzzi‐Perri *et al*. 2011; Yung *et al*. 2012; Sferruzzi‐Perri *et al*. 2013 *b*; Vaughan *et al*. 2015). Further work is required to determine the contribution of other environmental sensing pathways in the hypoxia/hypophagic‐induced alterations in placental transport phenotype (including the mechanistic target of rapamycin, general control non‐repressed 2, glucokinase, G‐protein coupled‐receptors 40 and 120, hypoxia‐inducible factors and AMPK) (Efeyan *et al*. 2015).

In summary, the mouse placenta adapts to help maintain fetal growth in response to moderate but not severe hypoxia. With moderate hypoxia at 13% O_2_, there were several morphological adaptations in the placenta beneficial to fetal growth, irrespective of the stage of pregnancy, which were related to changes in *Igf2* gene expression and insulin‐IGF signalling proteins in the Lz. Functionally, there were changes in placental transport and metabolism of glucose in response to moderate hypoxia at D19, which may have spared oxygen for fetal use and provided additional substrate for glycolytic production of energy in the feto‐placental tissues when the capacity for oxidative metabolism was limited. With the more severe hypoxia at 10% O_2_, there were no beneficial adaptations in placental morphology, transport or *Igf2* gene expression. Indeed, all the changes in placental phenotype at this degree of hypoxia were detrimental to nutrient and oxygen transfer to the fetus which, together with the maternal hypophagia, resulted in severe fetal growth restriction that was four‐fold greater than seen with moderate hypoxia. Taken together, these observations suggest that the adaptations in placental phenotype induced by maternal hypoxia are influenced by a wide range of factors, including maternal nutrition, fuel reserves, endocrine status, actual oxygen availability and feto‐placental nutrient demands related to gestational age and conceptus mass. The placenta therefore integrates these multiple signals to optimize maternal resource allocation to the fetus with respect to the prevailing environment. However, further studies are required to establish the importance of other signalling pathways and differential regulation of Akt phosphorylation at its two phosphorylation sites with respect to environmental sensing and adaptation of the placenta.

## Additional information

### Competing interests

The authors declare that they have no competing interests.

### Author contributions

ANS‐P and ALF conceived the project. All authors helped carry out the experiments and analysed the results. JSH, ANS‐P and ALF wrote the manuscript, which was subsequently commented on by ORV and EFdeL. All authors have approved the final version of the manuscript and agree to be accountable for all aspects of the work. All persons designated as authors qualify for authorship, and all those who qualify for authorship are listed.

### Funding

This work was funded by BBSRC studentship and *in vivo* skills award to JSH and by a NHMRC CJ Martin Fellowship and a Centre for Trophoblast Research Next Generation Fellowship to ANS‐P.

## Supporting information


**Table S1**. Maternal biometry, haematocrit and concentrations of blood glucose and plasma hormones Maternal biometry, haematocrit and concentrations of blood glucose and plasma hormones on D16 and D19 of pregnancy in normoxic dams (21% atmospheric O_2_ content); hypoxic dams exposed for 5 days either to 13% O_2_ from D11 to D16 or D14 to D19 or to 10% O_2_ from D14 to D19; and normoxic dams pair‐fed to the food intake of the 13% H dams from D11 to D16 or of the 10% H dams from D14 to D19.
