# Supplementary material for: Placental phenotype and resource allocation to fetal growth are modified by the timing and degree of hypoxia during mouse pregnancy
Source: J Physiol. 2015 Oct 26;594(5):1341–56. doi: 10.1113/JP271057 (PMC4771776; doi:10.1113/JP271057)
Supplement: Supplementary file 1 — Table S1. Maternal biometry, haematocrit and concentrations of blood glucose and plasma hormones Maternal biometry, haematocrit and concentrations of blood glucose and plasma hormones on D16 and D19 of pregnancy in normoxic dams (21% atmospheric O2 content); hypoxic dams exposed for 5 days either to 13% O2 from D11 to D16 or D14 to D19 or to 10% O2 from D14 to D19; and normoxic dams pair‐fed to the food intake of the 13% H dams from D11 to D16 or of the 10% H dams from D14 to D19. [file TJP-594-1341-s001.docx]

**Supplementary Table 1:** Maternal biometry, haematocrit and concentrations of blood glucose and plasma hormones on day (D) 16 and D19 of pregnancy in normoxic (N) dams (21% atmospheric O_2_ content), hypoxic (H) dams exposed for 5 days either to 13%O_2_ from D11-D16 or D14-D19 or to 10%O_2_ from D14-D19 and in normoxic dams pair fed (PF) to the food intake of the 13%H dams from D11-D16 or of the 10%H dams from D14-D19.

|  | **Day 16** | | | **Day 19** | | | |
| --- | --- | --- | --- | --- | --- | --- | --- |
|  | **Normoxia** | **13%PF** | **13%H** | **13%H** | **Normoxia** | **10%PF** | **10%H** |
| **Maternal body weight (g)** | | | |  |  |  |  |
| D1 | 20.6 ± 0.7 | 20.8 ± 0.4 | 21.5 ± 0.4 | 19.7 ± 0.4 | 20.7 ± 0.4 | 21.0 ± 0.4 | 20.6 ± 0.3 |
| Total | 29.8 ± 0.7 | 29.2 ± 0.6 | 30.8 ± 0.6 | 32.8 ± 0.9 | **35.3 ± 1.0^a^** | **32.3 ± 0.9^ab^** | **30.1 ± 0.7^b^** |
| Carcass | **23.2 ± 0.5^ab^** | **22.0 ± 0.3^a^** | **23.8 ± 0.4^b^** | 22.6 ± 0.3 | **23.2 ± 0.5^a^** | **22.3 ± 0.8^ab^** | **20.5 ± 0.4^b^** |
| %non-uterine gain | **14.2 ± 3.0^a^** | **6.1 ± 0.9^b^** | **11.0 ± 1.6^ab^** | 14.9 ± 1.8 | **12.7 ± 2.3^a^** | **5.9 ± 3.9^ab^** | **-0.2 ± 1.1^b^** |
|  | | | |  |  |  |  |
| **Maternal organ weights** | | | |  |  |  |  |
| Liver (g)  % of carcass | 1.65 ±0.19  7.12 ±0.80 | 1.42 ± 0.06  6.41 ± 0.21 | 1.74 ± 0.03  7.39 ± 0.08 | 1.61 ± 0.04  7.12 ± 0.13 | **1.65 ± 0.06^a^**  **7.06 ± 0.19^a^** | **1.30 ± 0.06^b^**  **5.8 ± 0.25^b^** | **1.40 ± 0.04^b^**  **6.83 ± 0.12^a^** |
|  |  |  |  |  |  |  |  |
| Kidneys (mg)  % of carcass | 257.2 ± 9.1  1.11 ± 0.02 | 243.0 ± 5.0  1.10 ± 0.01 | 246.5 ± 4.1  1.04 ± 0.01 | 232.9 ± 4.4  1.03 ± 0.02 | **238.0 ± 17.0^ab^**  1.03 ± 0.07 | **247.3 ± 6.2^a^**  1.12 ± 0.05 | **198.6 ± 14.1^b^**  0.97 ± 0.07 |
|  |  |  |  |  |  |  |  |
| Heart (mg)  % of carcass | 126.2 ± 7.9  0.54 ± 0.02 | 116.0 ± 2.9  0.53 ± 0.02 | 121.1 ± 2.8  0.51 ± 0.01 | 115.0 ± 2.3  0.51 ± 0.01 | 117.6 ± 3.4  0.50 ± 0.02 | 126.7 ± 6.6  0.50 ± 0.04 | 121.7 ± 2.1  0.60 ± 0.01 |
|  |  |  |  |  |  |  |  |
| Fat (mg)  % of carcass | 54.0 ± 4.9  0.23 ± 0.02 | 35.5 ± 4.9  0.16 ± 0.03 | 69.9 ± 13.4  0.30 ± 0.06 | 56.2 ± 3.5  0.25 ± 0.02 | **62.9 ± 6.0 ^a^**  **0.27 ± 0.02^a^** | **30.9 ± 3.0^b^**  **0.14 ± 0.01^b^** | **37.3 ± 2.5^b^**  **0.17 ± 0.02^b^** |
|  |  |  |  |  |  |  |  |
|  |  |  |  |  |  |  |  |
| **Maternal biochemistry** | |  |  |  |  |  |  |
| Haematocrit (%) | **44.6 ± 0.3^a^** | **44.7 ± 1.2^a^** | **49.0 ± 0.6^b^** | **49.0 ± 0.7*** | **43.8 ± 0.5^a^** | Not determined | **50.6 ± 2.1^b^** |
| Glucose (mM) | **9.7 ± 0.6^a^** | **10.1 ± 0.6^a^** | **7.6 ± 0.5^b^** | 8.2 ± 0.3 | 7.9 ± 0.3 | 6.8 ± 0.6 | 7.0 ± 0.3 |
| Insulin (ng/ml) | 0.69 ± 0.33 | 0.11 ± 0.02 | 0.21 ± 0.06 | 0.42 ± 0.16 | 0.38 ± 0.13 | 0.53 ± 0.37 | 0.34 ± 0.10 |
| IGF-1 (pg/ml) | 448 ± 75 | 710 ± 104 | 541 ± 144 | 715 ± 56 | 457 ± 108 | 647 ± 82 | 643 ± 158 |
| Leptin (ng/ml) | **8.5 ± 1.3^a^** | **4.7 ± 0.5^b^** | **9.4 ± 1.1^a^** | 8.5 ± 0.8 | **8.9 ± 1.3^a^** | **3.6 ± 0.5^b^** | **4.6 ± 0.4^b^** |
|  |  |  |  |  |  |  |  |

Data are mean ± SEM. For biometry: D16N, n=10; D16 13%H, n=13; D16 13%PF, n=7: D19N, n=16; D19 13%H, n=15; D19 10%H, n=14; D19 10%PF, n=13. For biochemical parameters: n=5-7 for each group at each age. Statistically significant differences are shown in bold. On D16, different letters represent significant differences between groups by one-way ANOVA with Bonferroni *post hoc* tests (P<0.05). On D19, different letters represent significant differences between normoxic, 10%PF and 10%H groups by one-way ANOVA with Bonferroni *post* hoc tests (P<0.05) and * D19 13%H group is significantly different from the D19N group by Student’s t-test (P<0.05). IGF-1, insulin-like growth fact
